# Limited Impacts of Cover Cropping on Soil N-Cycling Microbial Communities of Long-Term Corn Monocultures

**DOI:** 10.3389/fmicb.2022.926592

**Published:** 2022-06-10

**Authors:** Nakian Kim, Chance W. Riggins, María C. Zabaloy, Sandra L. Rodriguez-Zas, María B. Villamil

**Affiliations:** ^1^Department of Crop Sciences, University of Illinois, Urbana, IL, United States; ^2^Centro de Recursos Naturales Renovables de la Zona Semiárida, UNS-CONICET, Departamento de Agronomía, Universidad Nacional del Sur, Bahía Blanca, Argentina; ^3^Department of Animal Sciences, University of Illinois, Urbana, IL, United States

**Keywords:** N cycle genes, *nosZ*, *amoA*, *nifH*, *nirK*, *nirS*, maize (*Zea mays* L.), N fertilization

## Abstract

Cover cropping (CC) is a promising in-field practice to mitigate soil health degradation and nitrogen (N) losses from excessive N fertilization. Soil N-cycling microbial communities are the fundamental drivers of these processes, but how they respond to CC under field conditions is poorly documented for typical agricultural systems. Our objective was to investigate this relationship for a long-term (36 years) corn [*Zea mays* L.] monocultures under three N fertilizer rates (N0, N202, and N269; kg N/ha), where a mixture of cereal rye [*Secale cereale* L.] and hairy vetch [*Vicia villosa* Roth.] was introduced for two consecutive years, using winter fallows as controls (BF). A 3 × 2 split-plot arrangement of N rates and CC treatments in a randomized complete block design with three replications was deployed. Soil chemical and physical properties and potential nitrification (PNR) and denitrification (PDR) rates were measured along with functional genes, including *nifH*, archaeal and bacterial *amoA*, *nirK*, *nirS*, and *nosZ-I*, sequenced in Illumina MiSeq system and quantified in high-throughput quantitative polymerase chain reaction (qPCR). The abundances of *nifH*, archaeal *amoA*, and *nirS* decreased with N fertilization (by 7.9, 4.8, and 38.9 times, respectively), and correlated positively with soil pH. Bacterial *amoA* increased by 2.4 times with CC within N269 and correlated positively with soil nitrate. CC increased the abundance of *nirK* by 1.5 times when fertilized. For both bacterial *amoA* and *nirK*, N202 and N269 did not differ from N0 within BF. Treatments had no significant effects on *nosZ-I*. The reported changes did not translate into differences in functionality as PNR and PDR did not respond to treatments. These results suggested that N fertilization disrupts the soil N-cycling communities of this system primarily through soil acidification and high nutrient availability. Two years of CC may not be enough to change the N-cycling communities that adapted to decades of disruption from N fertilization in corn monoculture. This is valuable primary information to understand the potentials and limitations of CC when introduced into long-term agricultural systems.

## Introduction

As one of the most promising conservation practices, cover cropping (CC) bolsters the natural functions of an agricultural system through ecological intensification (Tittonell, 2014). This leads to various benefits like reducing soil erosion, returning organic matters to the soil, and weed suppression (Blanco-Canqui et al., 2015; Blesh et al., 2019; Reiss and Drinkwater, 2020). Many of such benefits contribute toward soil health, the soil’s capacity to support ecological services crucial for sustainable agriculture (Lehmann et al., 2020). Thus, CC has been widely proposed as a crucial strategy to improve the soil health of intensely managed systems that are vulnerable to soil degradation and nutrient pollution (IL-EPA et al., 2015). Simplified cropping systems concentrated on commercially important crops like corn [*Zea mays* L.] and soybean [*Glycine max* (L.) Merr.] dominate the crucial agricultural regions like the United States Midwest (Hatfield et al., 2018). As these systems, especially those based on corn, rely on frequent and heavy N fertilization, an estimated four Tg of inorganic N is applied each year in this region alone (USDA-ERS, 2020; Socolar et al., 2021). However, the low N use efficiency of these systems leads to excess soil N, which causes chemical imbalances that not only degrade the soil health but also become major sources of water pollution and greenhouse gas (GHG) emissions (Rengel, 2011; Fagodiya et al., 2017). Here, replacing bare fallows with CC can scavenge the excess N and release them slowly upon decomposition, thereby reducing the soil N in forms vulnerable to loss Indeed, past primary research and research syntheses have demonstrated that CC mixtures that include non-legumes generally reduce NO_3_^–^ leaching (Thapa et al., 2018) and nitrous oxide (N_2_O), a potent GHG, and emissions (Basche et al., 2014; Muhammad et al., 2019).

The soil microbes are the fundamental drivers of soil processes that constitute ecological services. Likewise, the soil N-cycling is largely determined by the complex web of activities by the N-cycling microbial communities. Therefore, changes in the soil environment from management practices can perturb the N-cycling communities and their functionality (Behnke et al., 2022; Kim et al., 2022b). Indeed, soil chemical imbalances and N losses in vulnerable agricultural systems are symptoms of excessive N fertilization disturbing the soil microbial N-cycling (Hirsch and Mauchline, 2015; Li et al., 2022). Likewise, various effects of CC, including excess soil N removal, organic matter return, and root exudation may also modify the soil environment and lead to unintended responses from the N-cycling communities. For instance, compared to leaving the terminated CC residues on the surface, incorporating them into the soil can rather increase N_2_O emissions by accelerating the N mineralization by the decomposers (Muhammad et al., 2019). Also, Foltz et al. (2021) have shown that although cereal rye CC decreased field N_2_O emission from a corn-soybean rotation, it increased the N_2_O production potential under assay settings, suggesting that CC might increase emissions without proper nutrient management. Thus, evaluating the effectiveness of CC in mitigating the detriments of excessive N fertilization must include how the soil N-cycling communities respond to changes in the soil environment under CC.

Abundances of the functional genes that code N-cycling enzymes represent the population sizes of their respective N-cycling communities. Indeed, the abundances of N-cycling functional genes tend to highly correlate with their respective inorganic N products (Lourenço et al., 2022). For example, N_2_O emission correlates negatively with functional genes that code N_2_O reductase but does so positively with those of nitrifiers (You et al., 2022). The commonly used N-cycling functional genes include *nifH* (nitrogenase; N-fixation), ammonia-oxidizing archaea (AOA) and bacterial (AOB) *amoA* (ammonia monooxygenase; nitrification), *nirK* and *nirS* (nitrite reductase; denitrification), and *nosZ-I* (nitrous oxide reductase; denitrification; Hirsch and Mauchline, 2015). As N fertilizers directly interact with the microbes harboring these genes, many studies have reported their sensitivity to N fertilization. For example, an Illinois study on the bioenergy production system of Mollisols reported that N fertilization (112 kg N/ha) increased the abundances of AOB *amoA*, but decreased those of *nirS* and *nifH* (Kim et al., 2022a). Meanwhile, a meta-analysis by Ouyang et al. (2018) compiled the results from 47 primary studies on how N fertilization affects these genes. The authors suggested that N inputs can intensify microbial N-cycling. Furthermore, the responses of these genes were sensitive to cofactors like fertilizer forms (organic vs. inorganic) and cropping systems (monoculture vs. rotation). Like these cofactors, CC might also alter the interactions between these functional genes and N fertilization. While these functional genes provide crucial insights into specific N-cycling communities, they do not equate to overall functionality as population size may not necessarily translate to activity. Thus, they need to be complemented by more direct measures of functionality like potential nitrification (PNR) and denitrification (PDR) rates determined by enzyme assays. Indeed, reports from various systems show that functional genes do correlate with PNR and PDR (Xu et al., 2012; Chen et al., 2013; Li et al., 2022). For instance, a study in acidic soils under soybean-corn-corn rotation reported significant correlations between *amoA* genes and PNR (Xu et al., 2012). However, primary research that coupled functional genes and more direct measures functionality is scarce for N-cycling communities under CC, especially within typical Midwestern cropping systems.

A few past studies have characterized the N-cycling functional genes under CC. For example, Hu et al. (2021), on cotton monoculture on Tennessee Alfisols, compared bacterial functional genes among bare fallow, legume CC, and grass CC between two N rates (0 and 67 kg N/ha), and between tillage and no-till. The authors reported that AOB *amoA* increased in abundance with legume CC and more so with N input, while grass CC only increased AOB *amoA* when unfertilized. They also showed that *nifH* increased sequentially from bare fallow, legume CC, and grass CC. Yet, the N rates of this study are much lower than those typically simplified cropping systems. Another recent study by Castellano-Hinojosa et al. (2022) also reported that CC, especially a legume and non-legume mixture, generally increased the abundance of the N-cycling functional genes. Yet, their system was a citrus orchard, which is also different from the United States Midwestern cropping systems. Thus, currently available studies on this topic are done in systems dissimilar to the intensely managed and simplified cropping systems typical to the major agricultural regions like the United States Midwest. Therefore, there is a critical lack of primary information on how the N-cycling functional genes respond to introducing CC to such systems.

Three past reports exist on this study’s experimental site where Kim et al. (2022b) and Huang et al. (2019) each characterized its soil and the N-cycling functional genes, respectively, before CC; while Kim et al. (2022c) described the soil microbiota with high taxonomic resolution using genus-level bioindicators after introducing CC. Kim et al. (2022b) demonstrated that significant soil acidification, increase in NO_3_^–^, and nutrient depletion marked the long-term effects of heavy N inputs in corn monocultures over resilient Mollisols. Huang et al. (2019) found that N fertilization decreased the abundance of *nifH* while increasing that of AOB *amoA*. Kim et al. (2022c) observed more diversified N-cycling communities in unfertilized soils, including an increased abundance of an indicator of AOA. They also reported soil acidification as the major modulating factor for the soil microbes of this system. Meanwhile, CC increased the abundances of N-fixers and *nirK* denitrifiers, while decreasing those of N_2_O reducers (Kim et al., 2022c). These potential shifts in the N-cycling guilds after introducing CC may have translated into the abundance of the functional genes, and, ultimately, into soil microbial N-cycling.

Thus, we aimed to investigate whether CC can mitigate the disruptions in the soil microbial N-cycling after long-term N fertilization in typical simplified agricultural systems. In accordance with aforementioned past reports on this study’s site, we hypothesized that: (i) N fertilization would decrease the number of *nifH* and AOA *amoA* but increase that of AOB *amoA*; (ii) introducing CC will increase the abundances of *nifH* and *nirK*; and that (iii) these changes will translate to changes in PNR and PDR. Our objective was to characterize the soil N-cycling communities after introducing CC to a long-term corn monoculture of 36 years of consistent management with and without N fertilization, using six commonly used functional genes (*nifH*, AOA and AOB *amoA*, *nirK*, *nirS*, and *nosZ-I*). This study also determined the soil properties, and PNR and PDR to test whether these genes are consistent with the changes in the soil environment and functionality by treatments. This study will provide primary information on how CC affects the soil microbial N-cycling in a simplified cropping system under heavy N inputs. This will be valuable for future research and decision-makers to evaluate CC as a strategy to improve soil health and nutrient cycling.

## Materials and Methods

### Experimental Site Description and Management Practices

**FIGURE 1:**
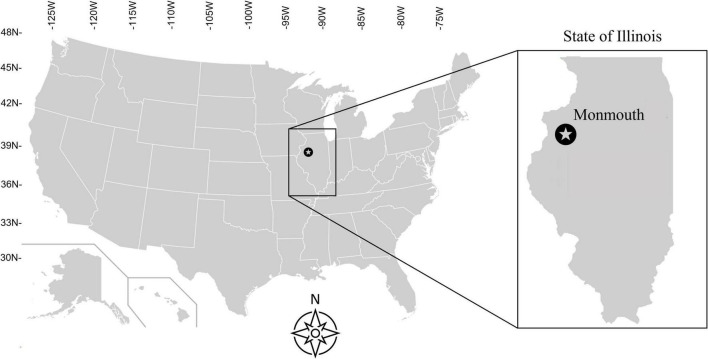
Location and coordinates of the experimental site within the United States and the state of Illinois.

The field experimental site was established at the Northwestern Illinois Agricultural Research and Demonstration Center (40° 55′ 50″ N, 90° 43′ 38″ W) in 1981 to study the effects of N fertilization rates (Nrates) on corn yields of continuous corn and corn-soybean short rotations (Figure 1). The mean annual precipitation and temperature of this site are 914 mm and 10.6°C, respectively, Illinois State Water Survey (2010). The soil series is Muscatune silt loam (fine-silty, mixed, and mesic Aquic Argiudoll), dark-colored and very deep soils of moderate permeability, and low surface runoff potential that developed under prairie vegetation in a layer of 2–3 m thick loess over glacial till (Soil Survey Staff et al., 2020). The topography of this site is nearly flat (Soil Survey Staff et al., 2020). Kim et al. (2022b) described further information on this experimental site and field management up to 2018.

This study started in 2018 and focused on introducing CC to the corn monoculture plots, spanning two CC growing seasons in 2018–2019 and 2019–2020. The experiment was arranged in a split-plot of Nrates (N0, N202, and N269; kg N/ha) and CC (cover crop, CC; bare fallow control, BF) in a randomized complete block design with three replicates. The main plot dimension was 18 m × 6 m, and the subplots were 18 m × 3 m. Fertilizer, weed, and pest management decisions were based on the best management practices as recommended by the Illinois Agronomy Handbook (Fernández and Hoeft, 2009). No P and K fertilizers or lime were applied during this experiment. Corn was harvested in mid-October with a plot combine (Almaco, Nevada, IA, United States). Following corn harvest, on October 3, 2018, and on October 19, 2019, a mixture of cereal rye [*Secale cereale* L.] and hairy vetch [*Vicia villosa* Roth.] was no-till drill-seeded at 84 kg seeds/ha (70% cereal rye, and 30% hairy vetch). Cover crop termination occurred in early May, both years following soil sampling, using glyphosate [*N*-(phosphonomethyl)glycine] (Roundup WeatheMAX^®^, Bayer AG, Leverkusen, Germany) at the rate of 1.89 kg ai/ha. Spring N fertilizer treatments were applied as incorporated urea ammonium nitrate (UAN, 28%) on May 15, 2019 and May 5, 2020, following CC suppression and before planting corn. Spring tillage using a rotary tiller (Dyna Drive Cultivator, EarthMaster, Alamo Group, Inc., Seguin, TX, United States) was conducted on June 3, 2019 and May 11, 2020. Corn cash crop was planted on June 3, 2019 and May 26, 2020, at 88,000 seeds/ha.

### Soil and Cover Crop Biomass Sampling and Properties Measurement

Soil sampling occurred on April 26, 2019 and April 30, 2020. Within each experimental unit, three composited soil subsamples, each up to 500 *g*, were taken from random points at 0–10 cm depth with an Eijelkamp grass plot sampler (Royal Eijkelkamp Company, Giesbeek, Netherlands) for microbial DNA extraction and PNR and PDR rates. These soil samples were transported in coolers filled with ice from the experimental site and then stored at −20°C. Also, three 0–90 cm depth soil core subsamples were taken per experimental unit using a tractor-mounted soil sampler with soil sleeve inserts (Amity Tech, Fargo, ND, United States). These soil cores were divided into depths of 0–30, 30–60, and 60–90 cm, and transported to the lab to determine the water content (%) and bulk density (Bd; Mg/m^3^). Only the data from 0 to 30 cm is reported in this study. About 10 *g* of soil per subsample was oven-dried at 105°C to measure the gravimetric water content and obtain Bd using the core method (Blake and Hartge, 1986). About 4 *g* of each subsample was used to determine water soil aggregate stability (WAS,%) with a west sieving apparatus (Eijkelkamp, Giesbeek, Netherlands). The remaining was sent to a commercial laboratory (Brookside Laboratories, Inc., New Bremen, OH, United States) to determine additional soil properties using standard methods recommended for United States-North Central region^1^. Thus, soil organic matter (SOM,%) content was determined by loss-on-ignition at 360°C (Schulte and Hopkins, 1996). Soil available P was measured with Bray I extraction (Pbray, mg/kg), and the extractable elements, including sulfur, calcium, magnesium, sodium, and potassium (K, mg/kg), with the Mehlich 150 III method (Ziadi and Tran, 2008). The summation of these exchangeable cations was used to estimate the cation exchange capacity (CEC, cmol_*c*_/kg; Sumner and Miller, 1996).

The PNR and PDR were measured with the methods adapted from Drury et al. (2008) and Malique et al. (2019), respectively. For PNR, a mixture of 6 *g* of the soil subsample and 40 mL of working solution was activated for assay by shaking them for an hour in a 125 mL jar covered with parafilm with several holes, on a rotary shaker (Corning^®^ LSE™ orbital shaker, Corning, NY, United States) at 180 RPM at room temperature. After assay activation, 5 mL of initial aliquot was taken and it was centrifuged for 10 min at 9,000 RPM (Sorvall^®^ RC 5C Plus, Kendro Laboratory Products, Asheville, NC, United States) and the supernatant was transferred and stored frozen for further analysis. NO_3_^–^ and NO_2_^–^ were measured using SmartChem 200 (Westco Scientific Instruments, Inc., Danbury, CN, United States). For the dry mass of the soil used in this equation, the mass of 1 *g* of soil subsample was measured after drying it for 48 h in the DKN-810 Mechanical Convection Oven (Yamato Scientific America Inc., Santa Clara, CA, United States). For PDR, 10 *g* of soil and 20 mL of working solution were put in 125 mL jars with septa caps. The headspace was vacuumed and, then, refilled with 100 mL of N_2_ gas with a syringe; this process was repeated once. Then, 20 mL of acetylene was inserted into the jar to prevent N_2_O reduction, and a 15 mL initial gas sample was taken and replenished with 15 mL of N_2_ gas. The jar that was shaken by hand to initiate reaction was incubated in a shaker (Eberbach, Ann Arbor, MI, United States). Gas samples were taken three times in 30 min intervals, replenishing the jars with 15 mL N_2_ gas each time. The gas samples were analyzed in a gas chromatograph equipped with an electron capture detector and autosampler (Shimadzu GC-2014 and AOC 5000 Plus, Kyoto, Japan). The N_2_O concentrations were calculated with the equation used in Malique et al. (2019), but the PDR (ng N/g of dry soil hour) was calculated using the *t* = 0_*min*_ N_2_O concentration instead of that of *t* = 30_*min*_.

### Soil DNA Extraction and Real-Time Quantitative Polymerase Chain Reaction Analysis

Soil DNA was extracted from 0.25 *g* of each homogenized soil subsample, using PowerSoil^®^ DNA isolation kits (MoBio Inc., Carlsbad, CA, United States) according to the manufacturer’s instruction, and stored at −20°C. A Nanodrop 1000 Spectrophotometer (Thermo Fisher Scientific, Waltham, MA, United States) was used to test the quantity and quality of the extracted DNA. The DNA samples were sequenced using primers for the bacterial V4 region 16S rRNA and archaeal 16S rRNA, fungal internal transcribed spacer (ITS) region, nitrogenase *nifH*, bacterial and archaeal ammonia monooxygenase *amoA*, nitrite reductase *nirK* and *nirS*, and nitrous oxide reductase *nosZ-I*. For this, the DNA samples were sent to W.M. Keck Center for Comparative and Functional Genomic lab at the University of Illinois Biotechnology Center (Urbana, IL, United States) for Illumina MiSeq paired-end System (2 × 250 bp; Illumina, Inc., San Diego, CA, United States). The maximum sample DNA concentration was set to 50 ng/μL. The primer sets used for amplification were 515F (GTGYCAGCMGCCGCGGTAA) and 806R (GGACTACVSGGGTWTCTAAT) for the bacterial 16S rRNA gene (Fierer et al., 2005); 349F (GTGCASCAGKCGMGAAW) and 806R (GGACTACVSGGGTATCTAAT) for the archaeal 16S rRNA gene (Colman et al., 2015); 3F (GCATCGATGAA GAACGCAGC) and 4R (TCCTCCGCTTATTGATATGC) for the fungal ITS region (Crawford et al., 2012); CS1-*nifH*-PolF-124-for (TGCGAYCCSAARGCBGACTC) and CSs-*nifH*-PolR-118-rev (ATSGCCATCATYTCRCCGGA) for *nifH* (Poly et al., 2001); Cren*amoA*23f (ATGGTCTGGCTW AGACG) and Cren*amoA*616r (GCCATCCATCTGTATG TCCA) for AOA *amoA* (Tourna et al., 2008); *amoA*-1F-10-for (GGGGTTTCTACTGGTGGT) and *amoA*-2R-10-rev (CCCCTCKGSAAAGCCTTCTTC) for AOB *amoA* (Rotthauwe et al., 1997); CS1-*nirK*876-209-for (ATYGGCGGVCAYG GCGA) and CS2-*nirK*1040-197-rev (GCCTCGATCAGRTTRT GGTT) for *nirK* (Henry et al., 2004); CS1-*nirS*Cd3aF-211-for (AACGYSAAGGARACSGG) and CS2-*nirS*R3cd-199-rev (GASTTCGGRTGSGTCTTSAYGAA) for *nirS* (Kandeler et al., 2006); and CS1-nosZ1F-16-for (WCSYTGTTCMTCGACA GCCAG) and CS2-nosZ1R-15-rev (ATGTCGATCARCTGV KCRTTYTC) for *nosZ-I* (Henry et al., 2006).

The resulting pooled amplicons of template DNA from the Illumina MiSeq system from all samples were prepared into PCR mixtures by mixing 1 μL of the pooled amplicon, 25.8 μL of ddH_2_O, 10 μL of 5X GoTaq green reaction buffer (Promega Corp., Madison, WI, United States), 4 μL of dNTP at 2.5 mM, 5 μL of MgCl_2_ at 25 mM, 0.2 μL of GoTaq DNA polymerase (Promega Corp., Madison, WI, United States), and 2 μL of primers for each forward and reverse sequences for each of the nine target sequences described above. This procedure was repeated by replacing the pooled amplicon with water to make controls. The mixtures were amplified in PCR with BioRad T100 thermal cycler (Bio-Rad Laboratories, Hercules, CA, United States) with parameters of 95°C for 10 min, followed by 34 cycles of amplification (45 s at 95°C; 45 s at 58°C; 45 s at 72°C), and a final extension at 72°C for 10 min. The amplified mixtures were run in electrophoresis with 1.5% agarose gel containing GreenGlo™ Safe DNA dye (Denville Scientific, Inc. Metuchen, NJ, United States), so that each pure, amplified marker genes are contained in one of the wells. The thick bands in the gel visible under UV light at the respective molecular weights of each target gene were cut out. Then, DNA was extracted from the cut gels using Monarch^®^ Genomic DNA Purification Kit (New England Biolabs, Ipswich, MA, United States), following the manufacturer’s instructions. The resulting DNA samples were again checked for quantity and quality using Nanodrop 1000 Spectrophotometer. Then, to make the standard curve, aliquots of each target gene were taken in unique amounts that equalized their estimated gene counts to 1.51 × 10^10^ copies for each of the nine marker genes. The aliquots were mixed and topped to 500 μL with 1× TE buffer. Then, the aliquots were diluted in 10-fold series and used 10^8^ to 10^1^ dilutions, so that the range of dilution encompasses the possible DNA copies per sample. Finally, quantitative polymerase chain reaction (qPCR) plates were prepared, including 70 μL of primers for each of the nine marker genes, 10 μL for each dilution of the standard curve for each plate, and 10 μL of aliquots for each sample. The prepared plates were run for high-throughput qPCR in a BioMark HD™ System (Fluidigm Corporation, South San Francisco, CA, United States) at the W.M. Keck Center for Comparative and Functional Genomics at the University of Illinois Biotechnology Center.

### Statistical Analysis and Visualization

Linear mixed models were fitted using the GLIMMIX procedure in SAS software version 9.4 (SAS Institute, Cary, NC, United States) to determine the effects of Nrate, CC treatments (CC), and their interactions on the response variables: soil properties, the log_10_-transformed gene copy counts, and PNR and PDR (Littell et al., 2006). The Nrate, CC, and their interaction were considered fixed effects, while the blocks, years, and their interactions with the fixed effects were considered random terms in the analysis of variance (ANOVA). For any significant treatment effects on the response variables in ANOVA, their least-square means were separated by treatment levels, with the *lines* option and setting the probability of a type I error at α = 0.10. To assess the correlations among the soil properties, PNR, PDR, and the nine marker genes, their Spearman’s rank correlation coefficients were calculated using the R function *cor* with option method = “spearman.” Relationships with coefficients (Spearman’s rho, ρ) above |0.8| were considered “very strong,” those between |0.6–0.8| as “strong,” and those between |0.4–0.6| as “moderate,” using the ranges from Huang et al. (2019) and setting the Type I error rate at α = 0.05. Weak relationships between |0.2–0.4| were disregarded even if they were statistically significant, to make the analysis more selective. The Spearman correlation also included soil pH, NH_4_^+^, and NO_3_^–^ as their correlations with the functional genes and PNR and PDR represent new information not reported by Kim et al. (2022c). The ggplot2 package in R version 4.1.0 was used to create the figures (Wickham, 2016; R Core Team, 2019).

## Results

### Responses of Soil Properties and Potential Nitrification Rate and Potential Denitrification Rate to N Rate and Cover Cropping Treatments

Table 1 shows the estimated treatment means, the standard errors of the mean (SEM), sample size (*n*), and the results of mean separation procedures for each level of Nrate, CC, and their interactions for selected soil properties (CEC, SOM, Pbray, K, Bd, and WAS) at 0–30-cm depth. Table 1 also shows the degrees of freedom (df) and probability values (*p*-value) associated with the ANOVA for each source of variation. The ANOVA and mean separation results for Nrate and CC main effects and their interaction effects on PNR and PDR are also included in Table 1. This study detected statistically significant Nrate main effects on CEC (*p* = 0.0098), Pbray (*p* = 0.0209), and K (*p* = 0.0005). The estimated means for CEC decreased sequentially with higher N rates. The mean Pbray and K decreased significantly with N fertilization, by 75 and 31%, respectively. Meanwhile, PNR and PDR did not have any statistically significant treatment effects. No statistically significant CC main effect nor Nrate × CC interaction effect was detected.

**TABLE 1:** Estimated treatment means, standard errors of the mean values (SEM), and sample size (*n*) of selected soil chemical properties, including cation exchange capacity (CEC, cmol_*c*_/kg), soil organic matter (SOM,%), bulk density (Bd, Mg/m^3^), water aggregate stability (WAS,%), available phosphorus (Pbray, mg/kg), and extractable potassium (K, mg/kg), along with the potential nitrification (PNR, mg N/kg dry soil day), and denitrification (PDR, ng N/g dry soil hr) rates determined under the N fertilization (Nrate) and cover cropping (CC) treatments, and their interactions.

Treatment*	*n*	CEC	SOM	Bd	WAS	Pbray	K	PNR	PDR
**Nrate**									
0	12	19.8 a	3.91	1.22	80.7	56.7 a	199 a	50.8	0.96
202	12	17.0 b	4.16	1.28	81.4	17.0 b	142 b	28.6	0.87
269	12	15.4 c	4.65	1.18	78.9	11.4 b	133 b	23.1	0.61
SEM		1.25	0.31	0.05	6.12	8.15	15.8	13.2	0.32
**CC**									
BF	18	17.2	4.04	1.22	79.3	28.4	158	23.9	0.74
CC	18	17.6	4.44	1.23	81.4	28.4	158	44.4	0.89
SEM		1.23	0.27	0.05	5.95	5.54	15.2	10.6	0.30
**Nrate × CC**									
0BF	6	19.7	3.94	1.20	80.5	58.3	204	47.0	0.76
0CC	6	19.9	3.89	1.23	80.9	55.2	194	54.6	1.17
202BF	6	16.8	4.11	1.27	80.8	15.5	138	9.0	1.04
202CC	6	17.3	4.20	1.28	82.0	18.5	146	48.1	0.70
269BF	6	15.0	4.07	1.18	76.7	11.3	132	15.7	0.43
269CC	6	15.7	5.23	1.18	81.2	11.5	133	30.4	0.80
SEM		1.28	0.43	0.05	6.28	8.40	16.93	15.0	0.40
**Sources of variation**	**df**	**CEC**	**SOM**	**Bd**	**WAS**	**Pbray**	**K**	**PNR**	**PDR**
Nrate	2	*0.0098*	0.2997	0.2573	0.7338	*0.0209*	*0.0005*	0.3436	0.5711
CC	1	0.1906	0.2790	0.7142	0.4138	1.0000	0.9746	0.2549	0.6738
Nrate × CC	2	0.8046	0.3437	0.5962	0.4923	0.5861	0.5344	0.2844	0.4813

*The degrees of freedom (df) and the probability values (p-values) associated with the analysis of variance (ANOVA) results are shown below. For each treatment and within a given column, mean values followed by the same lowercase letter were not statistically different (α = 0.10).*

*Nrate treatment levels: 0, 202, and 269 kg N/ha. CC treatment levels: bare fallow controls (BF) and hairy vetch and cereal rye cover crop mixture (CC).

### Responses of N-Cycling Functional Genes to N Rate and Cover Cropping Treatments

**FIGURE 2:**
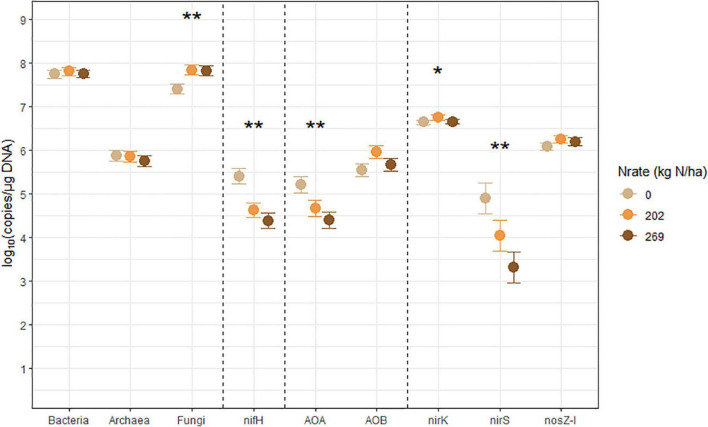
Treatment means of soil microbial marker genes (log_10_ copies/μg DNA), including bacterial (Bacteria) and archaeal (Archaea) 16S rRNA, fungal ITS region (Fungi), *nifH*, archaeal (AOA) and bacterial (AOB) *amoA*, *nirK*, *nirS*, and *nosZ-I* separated by N fertilization rate (Nrate), with their standard errors of the mean as whiskers. Asterisks indicate the level of significance associated with the probability value from analysis of variance of the factors (*p < 0.1, **p < 0.05). Nrate treatment levels were: 0, 202, and 269 kg N/ha.

**FIGURE 3:**
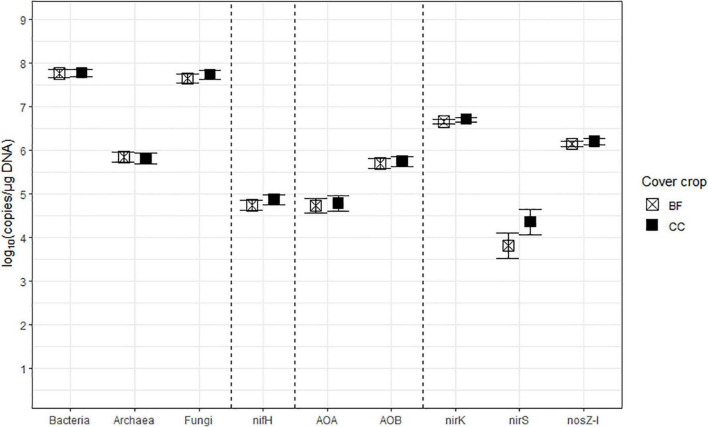
The treatment means of soil microbial marker genes in log_10_ (copies/μg DNA), including bacterial (Bacteria) and archaeal (Archaea) 16S rRNA, fungal ITS region (Fungi), *nifH*, archaeal (AOA) and bacterial (AOB) *amoA*, *nirK*, *nirS*, and *nosZ-I* separated by cover cropping (CC), with their standard errors of the mean as whiskers. The CC treatment levels were bare fallow control (BF) and hairy vetch and cereal rye cover crop mixture (CC).

**FIGURE 4:**
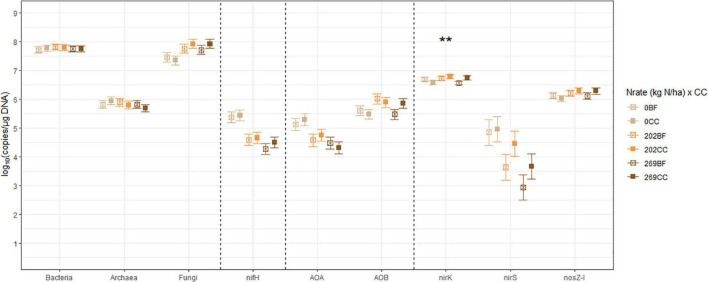
The treatment means of soil microbial marker genes in (log_10_ copies/μg DNA), including bacterial (Bacteria) and archaeal (Archaea) 16S rRNA, fungal ITS region (Fungi), *nifH*, archaeal (AOA) and bacterial (AOB) *amoA*, *nirK*, *nirS*, and *nosZ-I* separated by the interactions between N fertilization rate (Nrate) and cover cropping (CC), with their standard errors of the mean as whiskers. The asterisks indicate the probability value of the treatment effect from analysis of variance (**p < 0.05). Nrate treatment levels were: 0, 202, and 269 kg N/ha. CC treatment levels were bare fallow control (BF) and hairy vetch and cereal rye cover crop mixture (CC).

The estimated means, the SEM, and the mean separation by treatment levels of Nrate, CC, and their interactions for each log_10_-transformed count of the bacterial and archaeal 16S rRNA, fungal ITS, and the six N-cycling genes are summarized in Table 2. The sample size (*n*), df, and *p*-values associated with the results of ANOVA for each source of variation are also shown in Table 2. Figures 2–4 each illustrate the treatment means and the SEM of these genes for the Nrate main effect, CC main effect, and their interaction effect, respectively. The number of bacterial 16S rRNA region copies ranged between 1.95 × 10^7^ and 1.12 × 10^8^ copies/μg DNA, with the average count of 5.89 × 10^7^ copies/μg DNA. The number of archaeal 16S rRNA regions ranged between 2.04 × 10^5^ and 1.73 × 10^6^ copies/μg DNA, with the average count of 6.76 × 10^5^ copies/μg DNA. Bacterial and archaeal 16S rRNA did not have any treatment effect as a significant source of variation. The fungal ITS region counts ranged between 1.05 × 10^7^ and 1.70 × 10^8^ copies/μg DNA and averaged at 4.90 × 10^7^ copies/μg DNA. The fungal ITS region counts had a statistically significant (*p* = 0.0499) Nrate main effect, where the mean counts increased with N fertilization.

**TABLE 2:** Estimated treatment means and standard errors of the mean (SEM) of the log_10_-transformed copies (per μg DNA) of microbial marker genes, including bacterial (Bacteria) and archaeal (Archaea) 16S rRNA, fungal ITS region (Fungi), *nifH*, archaeal (AOA) and bacterial (AOB) *amoA*, *nirK*, *nirS*, and *nosZ-I* determined under N fertilization (Nrate), Cover crop (CC) treatments, and their interactions.

Treatment*	*n*	Bacteria	Archaea	Fungi	*nifH*	AOA	AOB	*nirK*	*nirS*	*nosZ-I*
**Nrate**										
0	12	7.75	5.88	7.41 b	5.40 a	5.21 a	5.54	6.64 b	4.90 a	6.07
202	12	7.81	5.85	7.84 a	4.62 b	4.66 b	5.95	6.76 a	4.04 ab	6.25
269	12	7.75	5.75	7.82 a	4.38 b	4.39 b	5.66	6.66 b	3.31 b	6.20
SEM		0.09	0.12	0.11	0.17	0.19	0.14	0.06	0.35	0.08
**CC**										
BF	18	7.76	5.84	7.65	4.74	4.72	5.70	6.66	3.81	6.15
CC	18	7.77	5.81	7.73	4.86	4.78	5.74	6.71	4.36	6.20
SEM		0.08	0.12	0.10	0.11	0.17	0.11	0.06	0.29	0.07
**Nrate × CC**										
0BF	6	7.72	5.82	7.46	5.37	5.13	5.60 abc	6.69 ab	4.84	6.12
0CC	6	7.78	5.94	7.35	5.44	5.28	5.48 bc	6.58 b	4.95	6.02
202BF	6	7.82	5.89	7.76	4.59	4.57	6.02 a	6.72 a	3.63	6.20
202CC	6	7.79	5.80	7.92	4.65	4.75	5.89 abc	6.79 a	4.45	6.29
269BF	6	7.75	5.82	7.71	4.26	4.47	5.47 c	6.56 b	2.94	6.11
269CC	6	7.75	5.69	7.93	4.50	4.31	5.85 ab	6.75 a	3.67	6.28
SEM		0.11	0.14	0.16	0.19	0.21	0.17	0.07	0.44	0.11
**Sources of Variation**	**df**	**Bacteria**	**Archaea**	**Fungi**	***nifH***	**AOA**	**AOB**	***nirK***	***nirS***	***nosZ-I***
Nrate	2	0.7685	0.3385	*0.0499*	*0.0278*	*0.0148*	0.2470	*0.0916*	*0.0363*	0.3361
CC	1	0.9054	0.5570	0.5333	0.2652	0.6824	0.7149	0.3611	0.1494	0.5367
Nrate × CC	2	0.8524	0.1309	0.5464	0.7333	0.3086	*0.1094*	*0.0404*	0.5373	0.4183

*The sample size (n), degrees of freedom (df), and the probability values (p-values) associated with the analysis of variance (ANOVA) results are shown below. For each treatment and within a given column, mean values followed by the same lowercase letter were not statistically different (α = 0.10).*

*Nrate treatment levels: 0, 202, and 269 kg N/ha. CC treatment levels: bare fallow controls (BF) and hairy vetch and cereal rye cover crop mixture (CC).

The nitrogenase-coding *nifH* gene counts were used to infer the abundance of the N-fixing community, and they ranged between 7.41 × 10^3^ and 7.41 × 10^5^ copies/μg DNA and averaged at 6.31 × 10^4^ copies/μg DNA. This gene had a statistically significant (*p* = 0.0278) Nrate main effect, where its means decreased with N fertilization. The AOA and AOB *amoA* gene counts were each used as proxies for the abundances of AOA and AOB communities, respectively. The AOA *amoA* counts ranged between 7.08 × 10^3^ and 6.46 × 10^5^ copies/μg DNA and averaged at 5.62 × 10^4^ copies/μg DNA. The bacterial *amoA* gene counts ranged between 3.98 × 10^4^ and 3.54 × 10^6^ copies/μg DNA, with an average of 5.25 × 10^5^ copies/μg DNA. AOA *amoA* had a statistically significant (*p* = 0.0148) Nrate main effect and decreased with N fertilization. Statistically marginal (*p* = 0.1094) N rate and CC interaction effects were detected for AOB *amoA*. Within BF, the mean abundance was greater with N202 than N269, with N0 being intermediate, while the means did not differ by N rate within CC; its mean count increased with CC when fertilized at N269. The *nirK* and *nirS* genes that code NO_2_^–^ reductase were both used as proxies for the NO_2_^–^-reducing denitrifier community. The *nirK* counts ranged between 2.51 × 10^6^ and 1.15 × 10^7^ copies/μg DNA, with an average of 4.79 × 10^6^ copies/μg DNA. This gene had a statistically significant (*p* = 0.0404) Nrate and CC interaction effect. The mean counts of *nirK* increased with N fertilization within CC, and they were greater with N202 than N269 within BF, with N0 being intermediate. The *nirS* count ranged between 81 and 3.89 × 10^5^ copies/μg DNA and had an average of 1.20 × 10^4^ copies/μg DNA. It had a statistically significant (*p* = 0.0363) Nrate main effect, so that its mean counts decreased sequentially with higher N rates, with N202 being intermediate. Finally, the N_2_O reductase coding *nosZ-I* gene, used to represent the N_2_O-reducing community, ranged between 6.03 × 10^5^ and 3.31 × 10^6^ copies/μg DNA with an average of 1.48 × 10^6^ copies/μg DNA. This gene did not have any statistically significant treatment effect.

### Correlations Among Gene Counts, Soil Properties, and Potential Nitrification Rate and Potential Denitrification Rate

**FIGURE 5:**
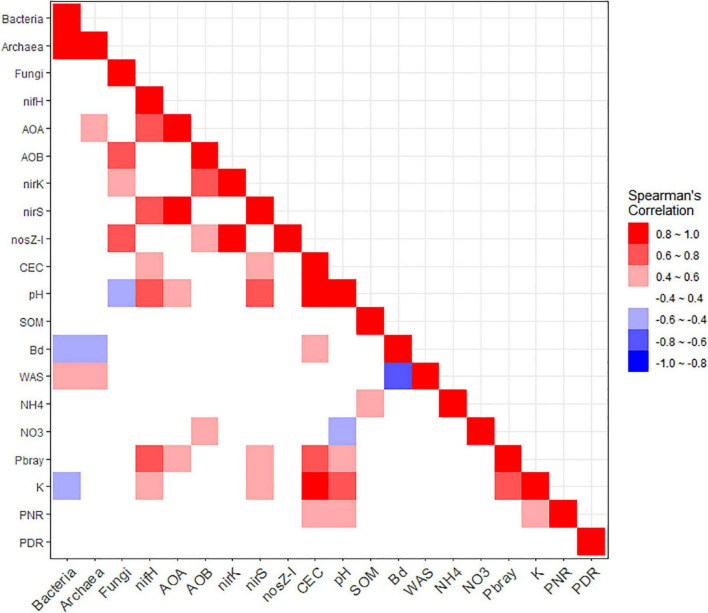
Heatmap depicting the matrix of Spearman’s rank correlation coefficients among the soil microbial marker genes, soil properties, and potential nitrification (PNR) and denitrification (PDR) rates. Marker genes included bacterial (Bacteria) and archaeal (Archaea) 16S rRNA, fungal ITS region (Fungi), *nifH*, archaeal (AOA) and bacterial (AOB) *amoA*, *nirK*, *nirS*, and *nosZ-I*. Soil properties included cation exchange capacity (CEC, cmol_*c*_/kg), pH, soil organic matter (SOM,%), bulk density (Bd, Mg/m^3^), water aggregate stability (WAS,%), ammonium (NH_4_, mg/kg), nitrate (NO_3_, mg/kg), available phosphorus (Pbray, mg/kg), and extractable potassium (K, mg/kg). The red and blue hues each indicate positive and negative associations, respectively. Higher color saturation indicates greater absolute values of Spearman’s rank correlation coefficients.

Supplementary Table 1 shows the Spearman’s rank correlation matrix with coefficients among bacterial and archaeal 16S rRNA, and fungal ITS regions, the six N-cycling functional genes, selected soil properties (CEC, pH, SOM, Bd, WAS, NH_4_^+^, NO_3_^–^, Pbray, and K), PNR, and PDR. This matrix is also visualized as a heatmap in Figure 5. We observed five very strong (|>0.8|), twelve strong (|0.6–0.8|), and 24 moderate (|0.4–0.6|) associations. The bacterial 16S rRNA gene counts had a very strong positive association with those of archaea, followed by moderate positive association with WAS and negative ones with Bd and K. Archaeal 16S rRNA associated moderately positively with archaeal *amoA* and WAS, and negatively with Bd. Fungal ITS region counts are associated strongly and positively with AOB *amoA* and *nosZ-I*, moderately positively with *nirK*, and moderately negatively with soil pH. The N-fixing *nifH* a had strong positive association with AOA *amoA*, *nirS*, pH, and Pbray, and moderately with CEC and K. AOA *amoA* had a very strong positive association with *nirS*, and moderate positive correlations with pH and Pbray. AOB *amoA* had strong positive associations with *nirK* and moderately positive associations with *nosZ-I* and NO_3_^–^. Denitrifying *nirK* gene counts are associated very strongly and positively with *nosZ-I*, while *nirS* is associated positively with pH strongly, and with CEC, Pbray, and K moderately. Besides the above-mentioned relationships, *nosZ-I* did not have further associations. Other than those already mentioned, CEC is very strongly and positively associated with pH and K, strongly with Pbray, and moderately with Bd and PNR. Soil pH is positively and strongly associated with K and moderately with Pbray and PNR, while having a moderate and negative association with NO_3_^–^. Meanwhile, SOM had a positive moderate association with NH_4_^+^. Bd had a very strong negative correlation with WAS. Pbray and K had a strong positive correlation, and K had a positive moderate association with PNR. In addition, there were 16 statistically significant (*p* < 0.05) but weak (*p* < |0.4|) correlations (Supplementary Table 1).

## Discussion

This study observed that CC had little impact on the soil environment of an intensely managed and simplified cropping system; thus, the soil properties remained stable since Kim et al. (2022b) before CC introduction. Higher N rates decreased CEC, which Kim et al. (2022b) explained, was due to the increasing soil acidity from the nitrifying N fertilizers and increased crop root uptakes (Tang and Rengel, 2003), leading to loss of exchange sites within soil particles (Barak et al., 1997). N fertilization also significantly depleted the soil available P and K *via* increased crop uptake. Before CC, Kim et al. (2022b) reported a statistically significant N rate main effect on Bd, but this study could not detect this. These contrasting results may owe to the thicker layer of topsoil analyzed in this study (30 cm) than in the previous study (15 cm). Similar to Kim et al. (2022b), SOM and WAS did not differ significantly by N rate. Indeed, Necpálová et al. (2014) estimated that statistically detectable changes in SOM of resilient Mollisols like in this study would take several decades. As for CC, only Kim et al. (2022c) found that CC decreased NO_3_^–^ in this experimental site. Similar results have been observed by Acuña and Villamil (2014), also in Illinois, where CC only affected NO_3_^–^ among various soil properties. Before CC, Kim et al. (2022b) demonstrated soil acidification and high nutrient availability as the main results of long-term N fertilization and corn monoculture over fertile United States Midwestern soils. Overall, in addition to the innate resilience of the Mollisols of this region, this study suggests that these soils may have become resistant to conservation practices after long-term exposure to disruptive practices. Thus, desired improvements from deploying CC may take more time and effort. The marginal CC effects on N-cycling communities and their functionality in this study may have stemmed from this resistant soil environment.

Consistent with Huang et al. (2019), this study found a statistically significant N rate’s main effect on fungal ITS counts, while bacteria and archaea did not respond to treatments. In both studies, fungal ITS region counts increased with N fertilization. This result agrees with the fungal species richness that also increased with N input in this site (Kim et al., 2022c). The fungal community may have benefited from N fertilization due to the stoichiometric differences between fungi and bacteria. Because fungal biomass has a greater C:N ratio than bacteria, fungi might benefit from N fertilizers increasing the returns of corn residues high in C:N ratio (Strickland and Rousk, 2010). Furthermore, fungi generally tolerate soil acidity better than bacteria, which allows them to take advantage of the acidifying soils from prolonged N fertilization (Strickland and Rousk, 2010; Kim et al., 2022c). Indeed, the ITS counts correlated negatively with soil pH. Another study in Illinois Mollisols on the effects of crop rotation and tillage also showed similar results, where only fungal abundance responded to crop rotation, due to soil acidification from corn monoculture (Behnke et al., 2020). Meanwhile, the bacterial and archaeal 16S counts had a strong monotonic relationship and correlated negatively and positively with Bd and WAS, respectively. Indeed, less dense soil and more stable soil aggregates are associated with higher soil carbon (C; Trivedi et al., 2017), wherein the soil’s microbial growth depends on (Kamble and Bååth, 2018). These results suggest that bacterial and archaeal abundances might depend more on these soil’s physical properties and perhaps soil C, than on N fertilizers and CC.

### Responses of the Soil N-Cycling Functional Genes

#### Detrimental Effect of N Fertilization on N-Fixing Community

As hypothesized, *nifH* decreased in abundance with N fertilization, consistent with the report before CC introduction (Huang et al., 2019). Similarly, a continuous wheat study of 22 years on the effects of N and P fertilization in Saskatchewan Mollisols also found that N fertilization decreased *nifH* and bacterial N-fixation (Li et al., 2020). Also, in a semi-arid grassland study in China, testing 3 years of N application (0, 25, 50, and 100 kg N/ha), the authors found that *nifH* initially increased in abundance at a low N rate but decreased sequentially as the N rate increased (Liao et al., 2021). High N availability from fertilizers mitigates the plant dependence on microbial N-fixers and disincentivizes their recruitment, which may explain why *nifH* decreased with higher N rates in this study (Liao et al., 2021). In addition, soil acidification is known to repress N-fixation (Bakari et al., 2020; Zhang et al., 2021), which also agrees with its strong positive association with soil pH. Therefore, soil acidification from excessive N fertilization could also be a factor. Kim et al. (2022c) reported that the genus *Mesorhizobium*, which includes nodule-forming N-fixers, increased in abundance with CC. Accordingly, this study hypothesized that this would reflect on the *nifH* counts. However, CC was not a significant source of variation for *nifH*. Indeed, *Mesorhizobium* also includes NO_2_^–^ reducers (Okada et al., 2005). Perhaps, Kim et al. (2022c) detected this genus not as an indicator of N-fixers but of denitrifiers. There have been studies reporting either positive (Castellano-Hinojosa et al., 2022) or insignificant (Hu et al., 2021) CC effects on *nifH*. The authors explained that the non-legume CC in the mixture depletes soil N, thus, encouraging N-fixation to meet the legume’s N demand (Castellano-Hinojosa et al., 2022). If this also applies to corn monoculture, this study might have not detected a significant CC effect on *nifH* because heavy fertilization overrode the N depletion by non-legume CC.

#### Ammonia-Oxidizing Archaea and Ammonia-Oxidizing Bacteria Responded Differently to Treatments (*amoA*)

In this study, a higher N rate sequentially decreased the abundance of AOA *amoA*. This agreed with Kim et al. (2022c), who also found that N fertilization decreased the abundance of *Nitrososphaera*, a genus, including neutrophilic AOA. Furthermore, AOA *amoA* correlated positively with soil pH. These results implied that AOA responded negatively to soil acidification, to which they may have contributed as nitrifiers themselves (Bolan and Hedley, 2003). Sun et al. (2019) studied the relationships between various soil properties and nitrifier communities in long-term fertilized, slightly acidic to neutral Chinese Vertisols (Wei et al., 2018). Similar to this study, the authors reported that AOA correlated the most with soil pH and decreased with soil acidification. AOA may adapt and form communities of distinct compositions at different soil pH (Hirsch and Mauchline, 2015). Thus, responses of these distinct AOA communities to changes in the soil conditions may vary by the initial soil pH they adapted to. For example, AOA is better adapted to acidic soils than AOB (Zhang et al., 2012) and can respond positively to N inputs in this condition (Gubry-Rangin et al., 2010). Yet, another AOA community that is initially adapted to more neutral soils may respond negatively to N inputs, like on Sun et al. (2019). Therefore, this study further suggested that soil acidity may have a detrimental impact on the AOA community that adapted to the slightly acidic to neutral soils typical to the US Midwest region. Meanwhile, CC did not have a significant impact on AOA *amoA*. This also agrees with Kim et al. (2022c), who reported no AOA, as indicators, is associated with CC. Castellano-Hinojosa et al. (2022) observed that legume and non-legume CC mixture increased AOA *amoA* in the citrus orchard, but this may not be the case for corn monoculture. A greenhouse study on Argentinian Mollisols by Allegrini et al. (2021) compared the nitrifier community responses to different CC termination methods. They found that AOA *amoA* increased with mechanical CC termination compared to chemical (glyphosate) termination and control without CC, which they explained that glyphosate can hamper the growth of AOA (Allegrini et al., 2021). Perhaps, chemical CC termination masked CC effects on AOA in this study as well, which might be worthwhile investigating in a field study.

According to the findings of Huang et al. (2019), this study hypothesized that AOB *amoA* will increase in abundance with higher N rates. Instead, the mean separations between fertilized and unfertilized control within CC were not statistically significant for AOB *amoA* in this study. Moreover, under bare fallow, AOB *amoA* mean counts did not differ statistically and significantly between fertilized plots and the unfertilized control. Overall, these results suggested that the AOB of this system was not as sensitive as AOA to N fertilization and its subsequent soil acidification. Indeed, past reports showed that AOB responds more sensitively to nutrient availability than soil pH. The meta-analysis by Ouyang et al. (2018) showed that AOB *amoA*, consistently, had positive effect sizes with N fertilization regardless of soil pH, while AOA *amoA* had an insignificant effect size below pH 6. Sun et al. (2019) also reported that soil acidification rather increased AOB abundance, although at the cost of their diversity. A microcosm study by Hink et al. (2018) showed that while AOA preferred a low rate of N inputs from organic sources, AOB increased in abundance with high rate of inorganic N inputs, showing that AOB may better exploit the inorganic N from fertilizers than AOA. Sun et al. (2019) suggested that the AOB community may occupy broader niches than AOA because they have more ecophysiological diversity. Perhaps, this ecological versatility allows AOB to adapt to acidifying soils and maintain their abundance. Also, Sun et al. (2019) showed that AOB significantly correlated with soil C. Thus, labile C from CC as root exudates and residues could explain why AOB *amoA* was more abundant with CC compared to bare fallow when fertilized at the highest N rate (Wang et al., 2021).

Despite a significant decrease in AOA *amoA* with a higher N rate, PNR did not differ by treatments in this study. While reports on PNR from similar cropping systems are scarce, a study from Northeastern Chinese Alfisols under soybean-corn-corn rotation found that PNR decreased with higher N rates (Xu et al., 2012). Another study from Kentucky Alfisols with corn monoculture and grass CC without bare fallow control found that PNR increase with N fertilization (Liu et al., 2017). Thus, currently, there is no consensus on how N fertilization affects PNR. The N rate effect on PNR may have not been statistically significant because stable AOB population size may have masked the decreasing contributions from AOA. Indeed, AOB *amoA* was 9.33 times more abundant than AOA *amoA* on average. Also, while AOA *amoA* abundance differed up to 15.13 times by treatment levels, AOB *amoA* only did so by 3.55 times. Likewise, the topsoil NO_3_^–^ level had a statistically significant positive correlation with AOB *amoA*, suggesting that AOB could have mainly driven the nitrification in this system. Meanwhile, there currently is no report on how PNR responds to CC in systems comparable to this study. While this study found no significant effects, further research will have to accumulate on this relationship.

#### Distinct Responses Between *nirK*- and *nirS*-Harboring Nitrite-Reducing Groups

In this study, *nirK* and *nirS* responded differently to the treatments. While N fertilization consistently decreased *nirS*, it slightly increased *nirK* within CC and had no difference from unfertilized control within bare fallow. These responses of *nirK* and *nirS* were analogous to AOB and AOA *amoA*, respectively, but the *nirK* differences by N rates within CC were statistically significant. Indeed, past reports agree with this study that N fertilization effects on *nirK* tend to range from insignificant to positive, while they are negative for *nirS* (Yin et al., 2014; Yang et al., 2017). For example, Yang et al. (2017) on a wheat-corn rotation system over alkaline soils of northern China reported that a higher N rate increased the abundances of *nirK* but decreased that of *nirS*. Yin et al. (2014) on soybean-corn-corn rotation observed that inorganic fertilizers decreased the abundances of *nirS* but had no impact on *nirK*. These genes may be responding differently to each other because the denitrifier guilds that each harbor adapt differently to soil acidification, similar to the *amoA*-nitrifiers discussed above. Indeed, this study observed that soil pH correlated strongly and positively with *nirS*, but not with *nirK*, which agreed with the past reports (Čuhel et al., 2010; Yang et al., 2017; Bowen et al., 2018). For example, Čuhel et al. (2010), on the effects of soil pH changes in pasture systems over Czech Mollisols, showed that *nirS* increased in abundance from acidic to alkaline soils, while *nirK* did not differ by soil acidity. Therefore, soil acidification from N fertilization in this study’s system may negatively impact the *nirS*-harboring denitrifiers. Also, Yang et al. (2017) showed that the abundance of *nirS* correlated significantly with the soil pH, while *nirK* did so with soil inorganic N levels. Thus, the *nirK*-harboring denitrifiers might be better adapted to N fertilizers, although this was only detected within CC in this study. Furthermore, fungi hold significant shares of the *nirK* community. For example, Xu et al. (2019) found that the fungal *nirK*-harboring community may contribute up to 50% of the N_2_O production during corn crops over black soils of Northeast China. Indeed, the present study observed statistically significant monotonic relationships among fungal ITS and *nirK*. As discussed earlier, fungi have better tolerance for soil acidity, which could have factored into *nirK* having less sensitivity to soil pH (Bowen et al., 2018).

This study hypothesized that CC would increase *nirK* but have no effect on *nirS*, based on the bioindicators of Kim et al. (2022c) that included *nirK*-harboring NO_2_^–^-reducers (*Mesorhizobium* and *Luteimonas*), but not *nirS* (Falk et al., 2010; Yin et al., 2014; Zhong et al., 2020). This hypothesis somewhat held true as *nirS* did not respond to CC, while *nirK* increased in abundance with CC under the context of N fertilization. However, past studies reported that CC tends to benefit both genes. The microcosm study by Wang et al. (2021) showed that ryegrass CC increased the abundances of *nirK* and *nirS* regardless of N rate (0 to 200 kg N/ha). They attributed these results to the labile C from CC root exudates and residues that generally promote the soil microbial community, including the denitrifiers. Similarly, Castellano-Hinojosa et al. (2022), on citrus orchards, found that CC increased the abundance of both genes. Still, their systems are very different from that of this study. Thus, the primary information on how *nirK* and *nirS* respond to CC within the typical corn-based cropping systems is critically lacking. In this study, *nirS* did have higher mean counts with CC than bare fallow control, but the CC main effect was still not statistically significant. Overall, this study found that *nirK* of this system may increase with CC when heavily fertilized, which also agreed with the bioindicators found by Kim et al. (2022c). However, whether *nirS* also responds positively to CC in this system will need further investigation.

Similar to nitrification, PDR did not have a statistically significant treatment effect, yet this result contrasts with past reports on PDR that increased with N fertilization and CC (Foltz et al., 2021; Li et al., 2022). Similar to *amoA* communities, *nirK* was much more abundant (nearly 400 times) than *nirS*, which decreased significantly with N fertilization. Also, *nirK* was more resistant to change than *nirS*, only differing up to 1.55 times in abundance by treatment levels compared to *nirS* that differed more than a hundred times. Maeda et al. (2017) reported that *nirK*-denitrifiers were more responsible for N_2_O emission in a compost pile despite their smaller number than *nirS*. Meanwhile, Liang et al. (2021) showed that denitrification was associated more with the *nirS* community because it was more responsive to climate factors and soil pH. Therefore, the lack of statistically significant responses from PDR could be reflecting both the dominant *nirK* contribution to PDR and sensitive changes in *nirS* communities to the treatments. Yet, this is beyond the insight that PDR can provide overall; moreover, nitrifier denitrification complicates this even more. Therefore, further investigation should determine how much each nitrifier and denitrifiers contributes to PDR, by using inhibitors of specific groups, for example.

#### N_2_O-Reducing Denitrifiers Unresponsive to N Fertilization and Cover Cropping (*nosZ-I*)

Consistent with past reports from this study’s site (Huang et al., 2019), the treatments had no statistically significant effect on *nosZ-I*. Conversely, Kim et al. (2022c) identified *Gemmatirosa* as a *nosZ*-harboring bioindicator that decreased with CC. Indeed, the denitrifiers in this genus belong to the *nosZ-II* clade (Xu et al., 2020), implying that *nosZ-I* and *nosZ-II* may respond differently to treatments in this system. This could be because these denitrifier groups each perform different soil functions and occupies distinct niches (Shan et al., 2021). Bowen et al. (2018) reported that the *nosZ-II* clade might be more sensitive to soil properties like pH than *nosZ-I*. Also, Wang et al. (2021) showed that the presence of ryegrass CC and high N rate increased the abundance of the *nosZ-I* clade, while *nosZ-II* did not respond to the treatments. Possibly, *nosZ-II* may have responded to N fertilizers and CC in this study, but this gene was not analyzed in this study. Therefore, future research should include *nosZ-II* for a more complete picture of how the N_2_O-reducers respond to CC in this system. Meanwhile, although high-rate N fertilization might not change the abundance of *nosZ*, soil acidification can interfere with the functionality of its product, N_2_O reductase, potentially exacerbating N_2_O emission (Qu et al., 2014). Thus, further investigation on the transcripts or enzyme activity of these genes might be necessary to determine how N_2_O reduction itself responds to CC in this system.

## Conclusion

This study is the first to determine how the soil N-cycling microbial community responds to cover crops in highly fertile, yet intensely manage cropping systems that are common to critical agricultural regions like the United States Midwest. Overall, the results on functional genes, the measured PNR and denitrification rates, and soil properties were consistent with each other without unexplainable discrepancies. Excessive N fertilization disrupts the N-fixer, AOA, and *nirS*-denitrifier communities, likely due to their sensitivity to soil acidification and a disincentivized plant-symbiosis for the N-fixers. Conversely, AOB and denitrifiers harboring *nirK* and *nosZ-I* responded subtly to N fertilization, likely because they are less sensitive to soil acidity or prefer high soil nutrient availability. Therefore, adaptation to soil acidification and nutrient availability from excessive N fertilization might be the discerning factor for the N-cycling communities in this system. Moreover, as in the cases of AOA *amoA* and *nirK*, some functional genes mirrored the previously reported genus-level bioindicators of the same site that are also known to harbor the respective genes. Thus, this study further supported the high-taxonomic resolution bioindicators as useful predictors of the soil microbial functions, although further investigation and reproduction of these results should follow. Despite these changes in the N-cycling communities, the enzyme assays did not respond to treatments, showing that overall functionality could be more resilient to changes. Contrary to the initial hypothesis, CC had a limited impact on the soil properties, N-cycling communities, and their functionality. This suggested that the soil environment and its N-cycling communities became resistant to changes after decades-long adaptation to consistent disruptions from heavy N fertilization and corn monocultures. Therefore, short-term CC may not be enough to improve heavily disrupted N-cycling communities. Further research should expand on this study with long-term CC, different cropping systems, and more functional genes for a more complete understanding of cover cropping as a sustainable practice to mitigate soil degradation and nutrient loss.

## Data Availability Statement

The original contributions presented in this study are included in the article/Supplementary Material, further inquiries can be directed to the corresponding author.

## Author Contributions

MV and NK: conceptualization, formal analysis. NK, CR, MZ, MV, and SR-Z: methodology. MV, SR-Z, and CR: resources. MV, MZ, CR, and NK: data curation. NK: visualization, writing—original draft preparation. MV and MZ: writing—review and editing. MV: supervision, project administration, and funding acquisition. All authors have read and agreed to the published version of the manuscript.

## Conflict of Interest

The authors declare that the research was conducted in the absence of any commercial or financial relationships that could be construed as a potential conflict of interest.

## Publisher’s Note

All claims expressed in this article are solely those of the authors and do not necessarily represent those of their affiliated organizations, or those of the publisher, the editors and the reviewers. Any product that may be evaluated in this article, or claim that may be made by its manufacturer, is not guaranteed or endorsed by the publisher.

## Footnotes

1 https://www.blinc.com/resources/testing-methods (accessed on 20 February 2022).

## Supplementary Material

Supplementary Table 1 Spearman rank correlation coefficients for bacterial and archaeal 16S rRNA, fungal ITS, *nifH*, AOA and AOB *amoA*, *nirK*, *nirS*, *nosZ-I*, estimated cation exchange capacity (CEC), pH, soil organic matter (SOM), bulk density (Bd), water aggregate stability (WAS), ammonium (NH_4_), nitrate (NO_3_), available phosphorus (Pbray), potassium (K), and potential nitrification (PNR) and denitrification (PDR) rates. Statistically significant coefficients (*p* < 0.05) are in bold. Cells are colored based on their coefficients, with red being positive and blue being negative, and darker the color the greater the value.
